# Optimization of Extraction Process for Total Flavonoids and Crude Polysaccharides from *Nephrolepis cordifolia* and Assessment of Their In Vitro Antioxidant Properties

**DOI:** 10.3390/molecules31162769

**Published:** 2026-08-09

**Authors:** Yuping Zhong, Xinran Xiong, Yunxuan Lv, Yongchang Chen, Zhiwei Liu, Xiaonan Zhang, Zuoliang Zheng

**Affiliations:** 1 School of Life Sciences, Jiaying University, Meizhou 514015, China; yupingzhong@jyu.edu.cn (Y.Z.);; 2 Guangdong Provincial Key Laboratory of Conservation and Precision Utilization of Characteristic Agricultural Resources in Mountainous Areas, School of Life Sciences, Jiaying University, Meizhou 514015, China

**Keywords:** *Nephrolepis cordifolia*, total flavonoids, crude polysaccharides, ultrasonic-assisted extraction, aqueous two-phase system, antioxidant activity

## Abstract

This study systematically optimized the extraction processes of two bioactive constituents, total flavonoids and crude polysaccharides, from the dried underground tubers of *Nephrolepis cordifolia* (L.) C. Presl, and further comprehensively characterized their in vitro antioxidant activities. For total flavonoid extraction, an ethanol–ammonium sulfate aqueous two-phase system (ATPS) was integrated with ultrasonic-assisted aqueous two-phase extraction (UATPE), and the process parameters were optimized via Box–Behnken design-based response surface methodology (RSM) using total flavonoid content (TFC) as the primary response indicator. The validated optimal conditions were determined as follows: extraction time of 40.67 min, solid-to-liquid ratio of 1:30.71 (g/mL), ammonium sulfate mass fraction of 24.03%, and ethanol concentration of 35% (*v*/*v*). Under these optimized conditions, the TFC reached (54.83 ± 1.58) mg rutin equivalents per gram dry weight (mg RE/g DW), which was significantly higher than the values obtained from conventional ATPS [(49.05 ± 0.41) (mg RE/g DW)] and traditional ethanol extraction [(28.68 ± 1.54) (mg RE/g DW)] (*p* < 0.05). For crude polysaccharide production, an ultrasonic-assisted water extraction followed by ethanol precipitation protocol was adopted, and parameters were screened through an L_9_ (3^3^) orthogonal experimental design. The obtained optimal extraction parameters were an extraction time of 10 min, three extraction cycles, and solid-to-liquid ratio of 1:15 (g/mL). A maximum crude polysaccharide yield of (14.89 ± 0.18) % was achieved under these conditions, with a relative standard deviation of 1.21% (*n* = 3) across parallel validation tests. Subsequent antioxidant assays demonstrated that the total flavonoid fraction from *Nephrolepis cordifolia* exhibited potent scavenging capacities against both 1,1-Diphenyl-2-picrylhydrazyl radical (DPPH•) and 2,2′-Azinobis-(3-ethylbenzthiazoline-6-sulphonate) radical cation (ABTS•^+^) radicals. Within the tested concentration range of 0.1–0.5 m g/mL, the crude polysaccharide presented a significant positive concentration-dependent enhancement of activity in four classical in vitro antioxidant systems, including DPPH radical scavenging, ABTS cation radical scavenging, hydroxyl radical scavenging, and total reducing power. Collectively, these findings confirm that the introduced ultrasonic-assisted extraction strategy remarkably improves the recovery efficiency of target bioactive components from *Nephrolepis cordifolia*. Both total flavonoids and crude polysaccharides display excellent in vitro free radical scavenging potential. This work provides a solid methodological basis for the efficient preparation of the two bioactive compounds, and highlights that *Nephrolepis cordifolia* is a promising candidate for further exploration as a natural antioxidant source.

## 1. Introduction

*Nephrolepis cordifolia* (L.) C. Presl, commonly known as “Stone Yellow Skin” (Shi Huang Pi), is a perennial fern that grows either epiphytically or terrestrially and belongs to the family Nephrolepidaceae [1]. It is widely distributed across the southeastern provinces of China. In traditional folk medicine, the rhizomes and petioles of this species have been used for centuries to clear heat, promote diuresis, alleviate coughing, and detoxify. Clinically, it has been traditionally employed in the management of fever, lung-heat-induced cough, orchitis, dysentery, enteritis, and otitis media [2,3]. Targeting the tubers of *Nephrolepis cordifolia* as the research material, modern phytochemical investigations have confirmed that this specific plant part contains a diverse array of bioactive constituents, including flavonoids, polysaccharides, and alkaloids. Among these, flavonoids constitute the principal pharmacologically active fraction, demonstrating well-documented antitumor, antioxidant, hepatoprotective, and immunomodulatory activities [4,5,6]. Nevertheless, prior research has predominantly focused on sterols and triterpenoids (such as β-sitosterol, oleanolic acid), while systematic phytochemical and pharmacological studies on flavonoids and polysaccharides remain comparatively limited [7]. This knowledge gap constrains the comprehensive development and rational utilization of *Nephrolepis cordifolia* as a medicinal plant resource.

An aqueous two-phase system (ATPS) is an efficient separation platform comprising two immiscible aqueous phases, typically formed by polymer–polymer or polymer–inorganic salt combinations. Target compounds are selectively partitioned between the two phases according to their differential partition coefficients, enabling mild, highly efficient, and single-step extraction with concurrent preliminary purification [8,9,10]. Ultrasonic-assisted aqueous two-phase extraction (UATPE) synergistically integrates the cavitation effects of ultrasound with the inherent advantages of ATPS. Specifically, microjets and shock waves generated during acoustic cavitation disrupt plant cell walls, thereby enhancing the solubilization and release of flavonoid compounds [11,12]. Concurrently, the gentle, aqueous environment of ATPS minimizes thermal degradation of heat-sensitive bioactive components, allowing simultaneous extraction and preliminary separation in a single operation. In contrast, conventional ethanol extraction, while featuring simple instrumentation and scalability for industrial applications, requires multiple extraction cycles and exhibits lower efficiency [13,14]. Water extraction followed by alcohol precipitation remains the most widely adopted method for recovering plant-derived polysaccharides, owing to its operational simplicity and cost-effectiveness [15,16]. Ultrasonic-assisted extraction markedly reduces extraction duration and enhances yield by rapidly disrupting plant cell walls via acoustic cavitation and localized high-pressure effects; it has thus been extensively applied in the isolation and preparation of diverse plant polysaccharides [17,18]. In this study, ultrasonic-assisted water extraction coupled with alcohol precipitation was employed for the efficient and environmentally sustainable recovery of crude polysaccharides from *Nephrolepis cordifolia*.

The in vitro antioxidant profile of the extracted flavonoids was systematically evaluated using four mutually complementary analytical methods: the 1,1-diphenyl-2-picrylhydrazyl radical (DPPH•) scavenging assay, the 2,2′-azinobis-(3-ethylbenzothiazoline-6-sulfonic acid) radical cation (ABTS•+) scavenging assay, the hydroxyl radical (•OH) scavenging assay, and the total reducing power assay [19]. This combined testing system eliminates the bias caused by a single antioxidant evaluation method, and delivers a comprehensive, multi-dimensional assessment of the intrinsic antioxidant potential of the target bioactive compounds. The rationale behind this targeted evaluation is threefold. Flavonoids and crude polysaccharides, two key bioactive components in this study, exhibit potent antioxidant activity that underpins their established health benefits [20,21]. Second, the selected complementary assays form a standardized multi-mechanism evaluation system covering radical scavenging, metal chelation and electron transfer, ensuring high result reliability and good cross-study comparability [19]. Third, this targeted evaluation strategy aligns well with the core objective of verifying the functional value of the optimized extraction process, and provides a direct experimental basis for subsequent applications in natural antioxidants and functional foods. This study aims to systematically optimize the extraction protocols for total flavonoids and crude polysaccharides from *Nephrolepis cordifolia*, and evaluate their in vitro antioxidant activities, so as to establish a solid scientific foundation and reliable technical reference for the integrated development of bioactive constituents from *Nephrolepis cordifolia*, the preparation of natural antioxidants, and the research and development of related functional food products.

## 2. Results and Discussion

### 2.1. Optimization of the Total Flavonoid Extraction Process

#### 2.1.1. Construction of the Ethanol–Ammonium Sulfate Phase Diagram

The phase diagram of the ethanol–ammonium sulfate system clearly delineates a biphasic region (upper right) and a monophasic region (lower left), separated by the binodal curve. Within the biphasic region, the salt-rich phase—characterized by higher density—forms the lower layer, whereas the ethanol-rich phase—exhibiting lower density—constitutes the upper layer. Target flavonoid compounds preferentially partition into the upper (ethanol-rich) phase, while macromolecular impurities, including proteins and polysaccharides, predominantly reside in the lower (salt-rich) phase. To ensure stable and reproducible phase separation, all subsequent single-factor and response surface methodology experiments were conducted exclusively within the biphasic region.

#### 2.1.2. Results of Single-Factor Experiments

**Figure 1:**
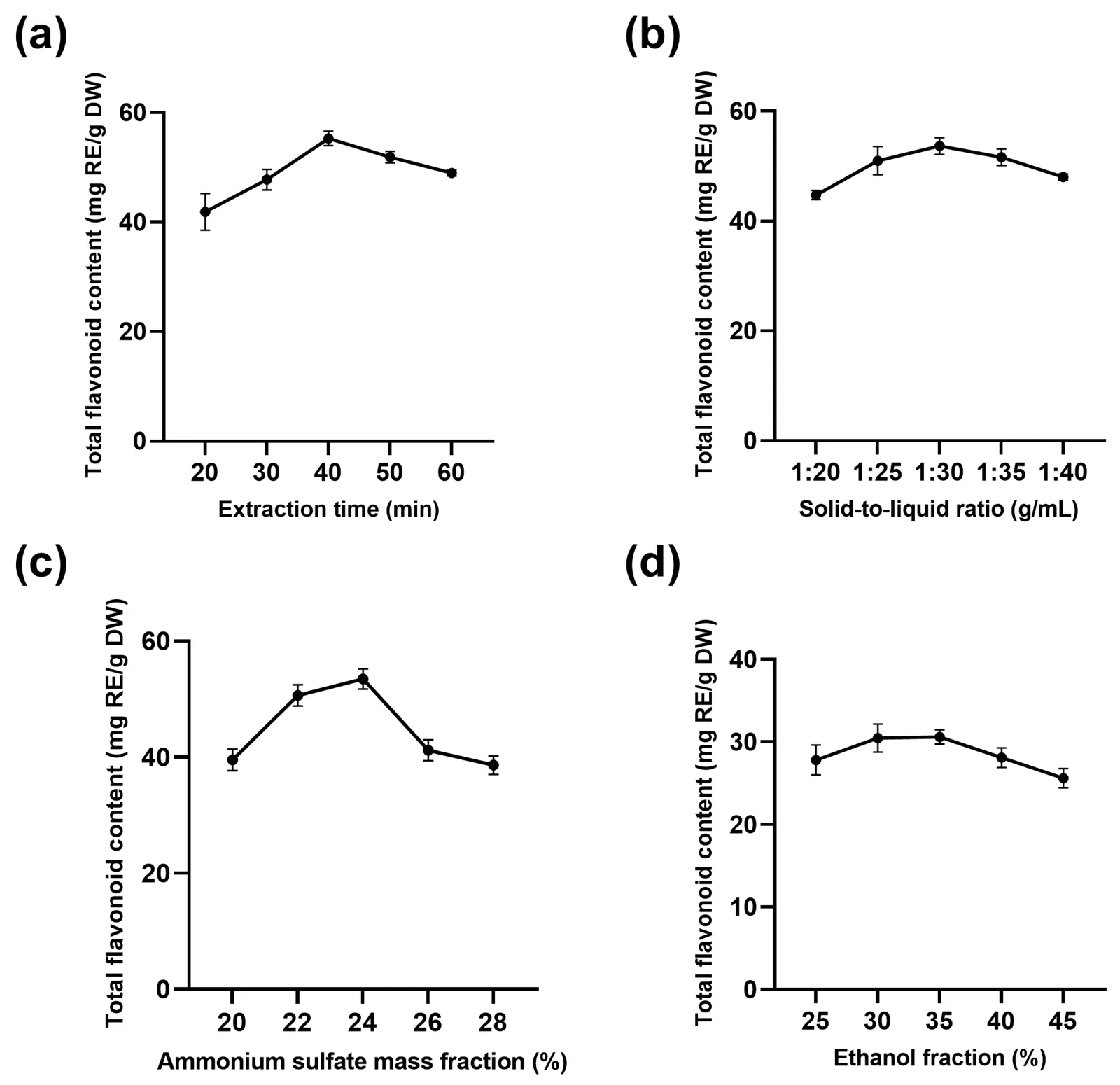
Single-factor results for flavonoid extraction parameters: Effects of 4 variables on total flavonoid content from *Nephrolepis cordifolia* via ultrasonic-assisted aqueous two-phase extraction: (**a**) extraction time; (**b**) solid-to-liquid ratio; (**c**) mass fraction of ammonium sulfate; (**d**) ethanol concentration.

As shown in Figure 1a, the total flavonoid content (TFC) exhibited an initial increase followed by a subsequent decrease with prolonged extraction time, attaining a maximum value at 40 min. Extended extraction durations promote co-extraction of impurities and accelerate oxidative degradation and structural breakdown of flavonoids, thereby diminishing overall extraction efficiency. Accordingly, 40 min was selected as the optimal extraction time. As illustrated in Figure 1b, TFC increased initially and then declined as the solid-to-liquid ratio was raised from 1:20 to 1:40 (g/mL), reaching a peak of 60.9 mg rutin equivalents per gram dry weight (mg RE/g DW) at a ratio of 1:30 (g/mL). A suboptimal solvent volume restricts solvent penetration into the matrix and limits flavonoid solubilization, whereas an excessive solvent volume compromises the stability of the two-phase system and enhances non-selective dissolution of interfering compounds. Hence, a solid–to-liquid ratio of 1:30 (g/mL) was adopted as the optimum. Figure 1c reveals a bell-shaped dependence of TFC on ammonium sulfate mass fraction within the range of 20–28%, with the maximum observed at 24%. An excessively high ammonium sulfate concentration disrupts the phase separation equilibrium and impedes the partitioning of flavonoids into the upper phase. Consequently, 24% was identified as the optimal ammonium sulfate mass fraction. As depicted in Figure 1d, flavonoid yield progressively increased with ethanol concentration rising from 25% to 35%, plateauing at 35%. Further elevation beyond this threshold reduces the polarity of the upper phase, thereby decreasing flavonoid solubility and extraction capacity. Thus, 35% ethanol was established as the optimal concentration.

#### 2.1.3. Response Surface Optimization Results

**Figure 2:**
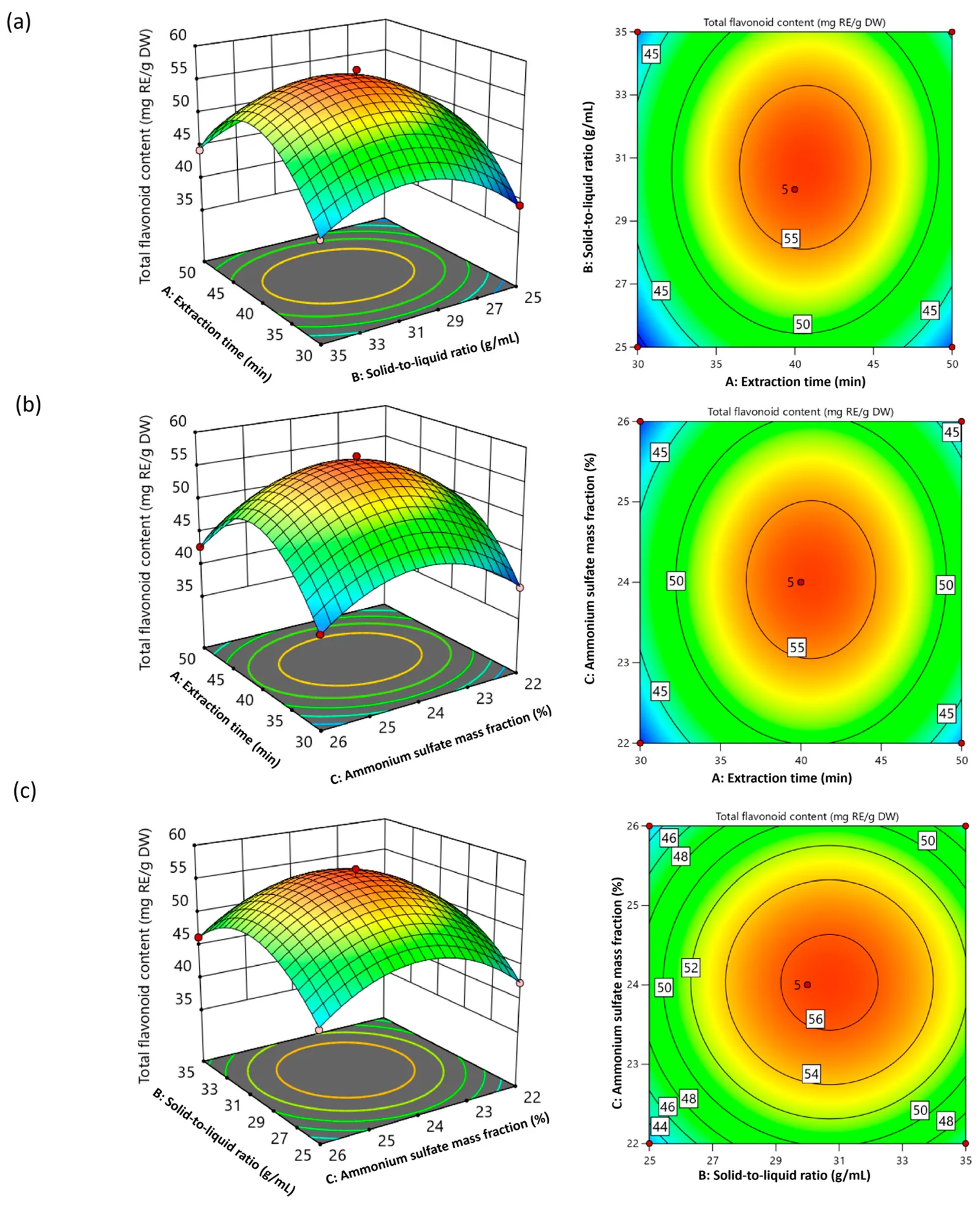
Three-dimensional response surface plots and corresponding contour plots for the Box–Behnken design: (**a**) extraction time × solid-to-liquid ratio; (**b**) extraction time × ammonium sulfate mass fraction; (**c**) solid-to-liquid ratio × ammonium sulfate mass fraction (generated by Design-Expert 13). The blue-to-red color gradient represents the increase of total flavonoid content (mg RE/g DW), and the colored spheres denote the actual experimental points.

Using extraction time (A), solid-to-liquid ratio (B), and ammonium sulfate mass fraction (C) as independent variables, a total of 17 experiments were conducted according to a Box–Behnken design (BBD) (Table 1). A quadratic polynomial regression model was developed to describe the relationship between these variables and TFC [Y, expressed in (mg RE/g DW)]: Y = 56.45 + 1.16A + 1.65B + 0.1987C + 0.375AB + 0.09AC − 0.0025BC − 9.15A^2^ − 5.94B^2^ − 6.16C^2^. Analysis of variance (ANOVA) results (Table 2) revealed that the regression model was highly significant (*p* < 0.0001), with a coefficient of determination (*R*^2^) of 0.9971 and an adjusted *R*^2^ of 0.9934, indicating an excellent fit to the experimental data. The lack-of-fit test was non-significant (*p* = 0.8500), and the coefficient of variation (*CV*) was only 1.22%, confirming the high precision and reproducibility of the experimental measurements. The interaction effects of AB, AC, and BC are not significant in the model (*p*-values are all greater than 0.05), indicating that the interactions between these factors have a relatively minor impact on the total flavonoid extraction yield. The quadratic terms A^2^, B^2^, and C^2^ exhibit highly significant effects (*p* < 0.0001), suggesting the presence of nonlinear relationships between each factor and the total flavonoid extraction yield. Based on standardized regression coefficients, the order of the relative influence of the three factors on TFC was as follows: solid-to-liquid ratio (B) > extraction time (A) > ammonium sulfate mass fraction (C). The response surface plots (Figure 2) visually illustrate the effects of various factors and their interactions on the total flavonoid extraction yield. It can be observed from the plots that the total flavonoid extraction yield initially increases and then decreases with increasing extraction time and solid–liquid ratio, which is consistent with the significance of the quadratic terms. The mass fraction of ammonium sulfate has a relatively minor impact on the total flavonoid extraction yield, showing little variation across different levels, which aligns with the results of the analysis of variance.

#### 2.1.4. Verification of Optimal Flavonoid Extraction Conditions

The optimal flavonoid extraction conditions, determined via response surface methodology (RSM), were as follows: extraction time = 40.67 min; solid-to-liquid ratio = 1:30.71 (g/mL); ammonium sulfate mass fraction = 24.03%; and ethanol concentration = 35% (*v*/*v*). Three independent extraction replicates yielded a mean TFC of 54.83 (mg RE/g DW)-close to the RSM-predicted value of 56.60 (mg RE/g DW)-with an absolute deviation of 1.77 (mg RE/g DW) and a relative standard deviation (*RSD*) of 3.13%. The strong agreement between experimental and predicted values confirms the high predictive accuracy and robustness of the established RSM model. Compared with conventional aqueous two-phase system extraction [(49.05 ± 0.41) (mg RE/g DW)] and conventional ethanol extraction [(28.68 ± 1.54) (mg RE/g DW)], the ultrasonic-assisted aqueous two-phase extraction (UATPE) method demonstrated marked improvements in extraction efficiency. These results clearly indicate that ultrasound irradiation synergistically enhances aqueous two-phase system (ATPS)-based flavonoid recovery from *Nephrolepis cordifolia*, consistent with prior reports on ultrasound-enhanced ATPS applications in phytochemical extraction [22].

We acknowledge that the current analysis, based solely on TFC, provides an incomplete picture of the phytochemical profile. While the results confirm the quantitative advantage of UATPE in total yield, future studies will employ liquid chromatography–tandem mass spectrometry analysis to identify and quantify the specific flavonoid compounds (e.g., quercetin, kaempferol, and their glycosides) present in the extracts obtained via UATPE, conventional ATPS, and ethanol extraction. This will elucidate whether the observed enhancement is accompanied by a qualitative difference in flavonoid composition, potentially leading to a more bioactive profile. Furthermore, measurement of total phenolic content will be conducted to evaluate the contribution of non-flavonoid phenolics to the overall antioxidant capacity, thereby providing a more comprehensive understanding of the extracts’ bioactivity. This planned phytochemical profiling represents a key direction for subsequent research to fully characterize the advantages of the UATPE method.

### 2.2. Optimization of Crude Polysaccharide Extraction Process

#### 2.2.1. Results of Single-Factor Experiments

**Figure 3:**
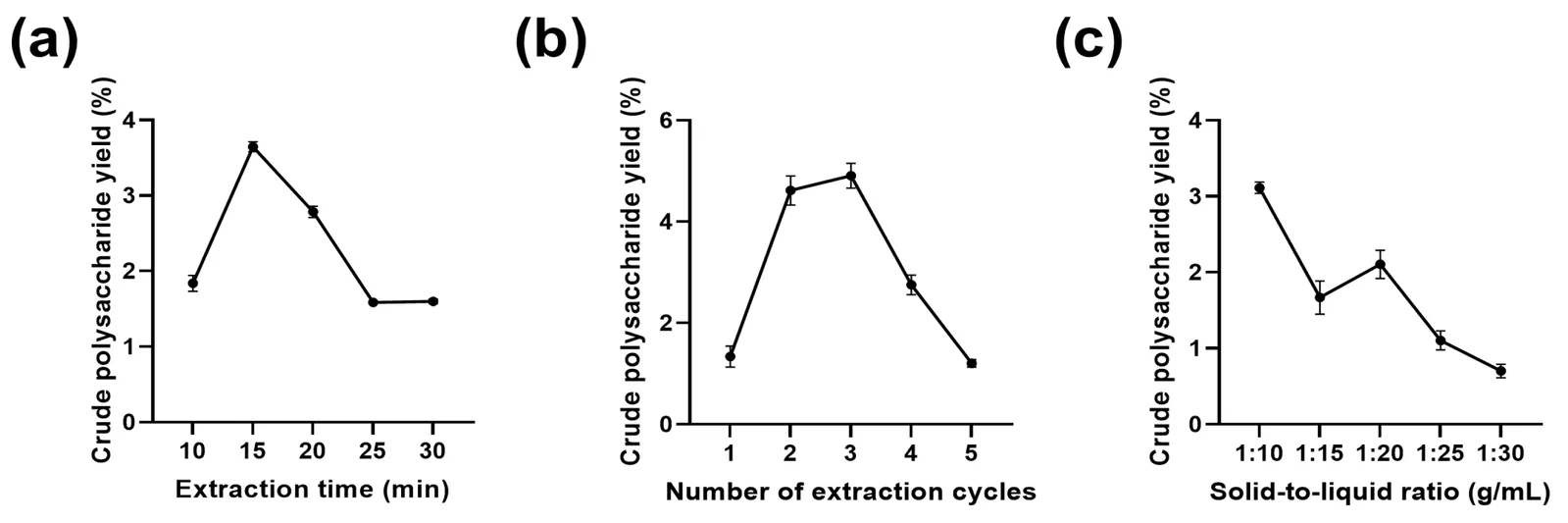
Single-factor experimental results for polysaccharide extraction parameters. The effects of individual variables on the crude polysaccharide yield from *Nephrolepis cordifolia* via ultrasonic-assisted water extraction and alcohol precipitation are presented as follows: (**a**) extraction time; (**b**) number of extraction cycles; (**c**) solid-to-liquid ratio.

As shown in Figure 3a, the yield of crude polysaccharides initially increased and then decreased with prolonged extraction time, reaching a peak of 3.65% at 15 min. Beyond 15 min, the sustained thermal and mechanical shear effects of ultrasound began to degrade polysaccharide molecules, leading to the cleavage of glycosidic bonds and increased dissolution of impurities, resulting in a decline in yield. Considering both extraction efficiency and energy consumption, 10, 15, and 20 min were selected as the levels for the orthogonal design. As shown in Figure 3b, the polysaccharide yield initially increased and then decreased with the number of extraction cycles, reaching a maximum of 4.91% at three cycles. Beyond three cycles, the continuous dissolution of non-polysaccharide impurities interfered with the determination, and energy consumption increased. Therefore, 2, 3, and 4 cycles were chosen as the levels for the orthogonal design. As shown in Figure 3c, the polysaccharide yield exhibited a pattern of first sharply decreasing, then slightly increasing, and finally decreasing again as the solid-to-liquid ratio increased. Although the apparent yield was highest (3.11%) at a ratio of 1:10 (g/mL), the limited solvent volume constrained the complete dissolution of polysaccharides. At a ratio of 1:20 (g/mL), polysaccharide dissolution was more complete, leading to a slight recovery in yield. Considering all factors, 1:10, 1:15, and 1:20 (g/mL) were selected as the levels for the orthogonal design.

#### 2.2.2. Orthogonal Optimization and Analysis of Variance

The results of the L_9_ (3^3^) orthogonal design showed that, based on range analysis (*R* values), the order of influence of the three factors on crude polysaccharide yield was as follows: number of extraction cycles (B, *R* = 3.86) > solid-to-liquid ratio (C, *R* = 3.15) > extraction time (A, *R* = 1.08). According to the mean values of each factor at each level (A: *k*_1_ *> k*_3_ *> k*_2_; B: *k*_2_ *> k*_3_ *> k*_1_; C: *k*_2_ *> k*_3_ *> k*_1_), the optimal combination was determined as A_1_B_2_C_2_ (i.e., extraction time 10 min, three extraction cycles, and solid-to-liquid ratio 1:15 (g/mL)). Using *F*_0.05_ (2, 2) = 19.00 as the threshold for ANOVA, the *F*-values of all three factors were lower than *F*_0.10_ (2, 2) = 9.00, indicating that within the studied experimental range, none of the factors reached a significance level of 0.05. However, the conclusions from range analysis and ANOVA were consistent. The model *R*^2^ = 0.838 provided statistical support for the determined optimal conditions.

#### 2.2.3. Verification of Optimal Crude Polysaccharide Extraction Conditions

Three independent extraction replicates were conducted under the optimal conditions (extraction time 10 min, three extraction cycles, solid-to-liquid ratio 1:15 g/mL). The mean crude polysaccharide yield was (14.89 ± 0.18) % (*RSD* = 1.21%), which was significantly higher than the maximum yield (4.80%) obtained in the single-factor experiments. High reproducibility and precision demonstrate that orthogonal optimization effectively enhances polysaccharide extraction efficiency via multi-factor synergistic interaction. Furthermore, the total extraction time (10 min × 3 cycles) was much shorter than that of conventional hot water extraction (typically 1–3 h), fully highlighting the time-and energy-saving benefits of ultrasonic extraction.

### 2.3. Evaluation of Antioxidant Activity of Nephrolepis cordifolia

#### 2.3.1. Antioxidant Activity of Total Flavonoids from *Nephrolepis cordifolia*

**Figure 4:**
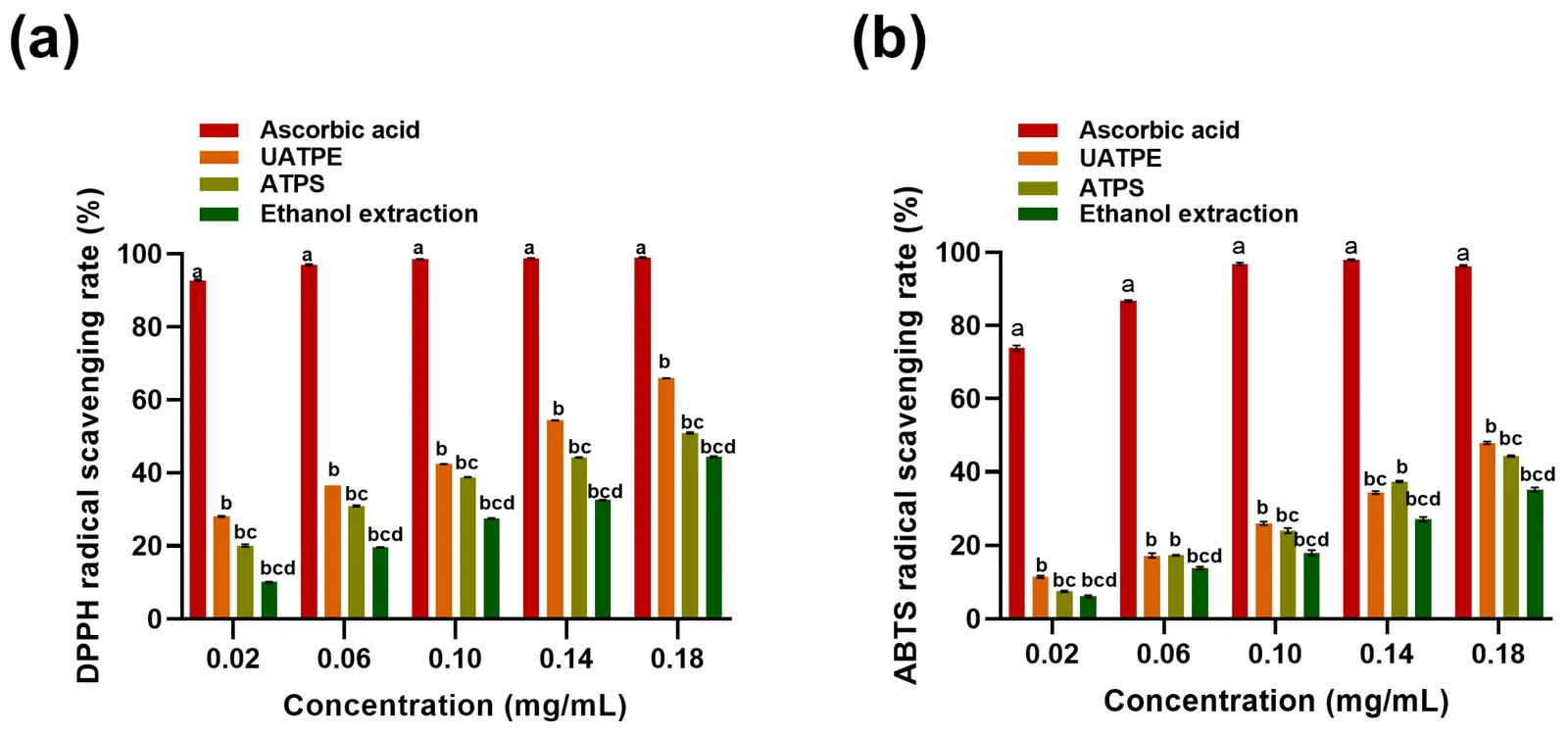
In vitro antioxidant activities of total flavonoids from *Nephrolepis cordifolia* evaluated via DPPH (**a**) and ABTS (**b**) radical scavenging assays. Three extraction groups (UATPE, ATPS, ethanol extraction) and ascorbic acid as the positive control were included in the test. Different lowercase letters indicate significant differences between groups at the same concentration (*p* < 0.05).

The DPPH and ABTS radical scavenging activities of total flavonoids obtained by the three extraction methods are shown in Figure 4. Regarding DPPH radical scavenging, the scavenging capacity of flavonoid extracts from all three methods increased in a dose-dependent manner with increasing concentration. At a flavonoid concentration of 0.18 mg/mL, the order of scavenging activity was ascorbic acid > UATPE > ATPS > ethanol extraction. For ABTS radical scavenging, within the concentration range up to 0.18 mg/mL, the order was ascorbic acid > UATPE > ATPS > ethanol extraction, with the scavenging rate continuously increasing with concentration. The flavonoids obtained by UATPE exhibited superior antioxidant activity in both DPPH and ABTS systems. This is attributed to the effective quenching of free radicals by the phenolic hydroxyl groups in flavonoid molecules, coupled with the good protection of bioactivity provided by the mild aqueous environment of ATPS, which corroborates the conclusions drawn from the established extraction process optimization.

#### 2.3.2. Antioxidant Activity of Crude Polysaccharides from *Nephrolepis cordifolia*

**Figure 5:**
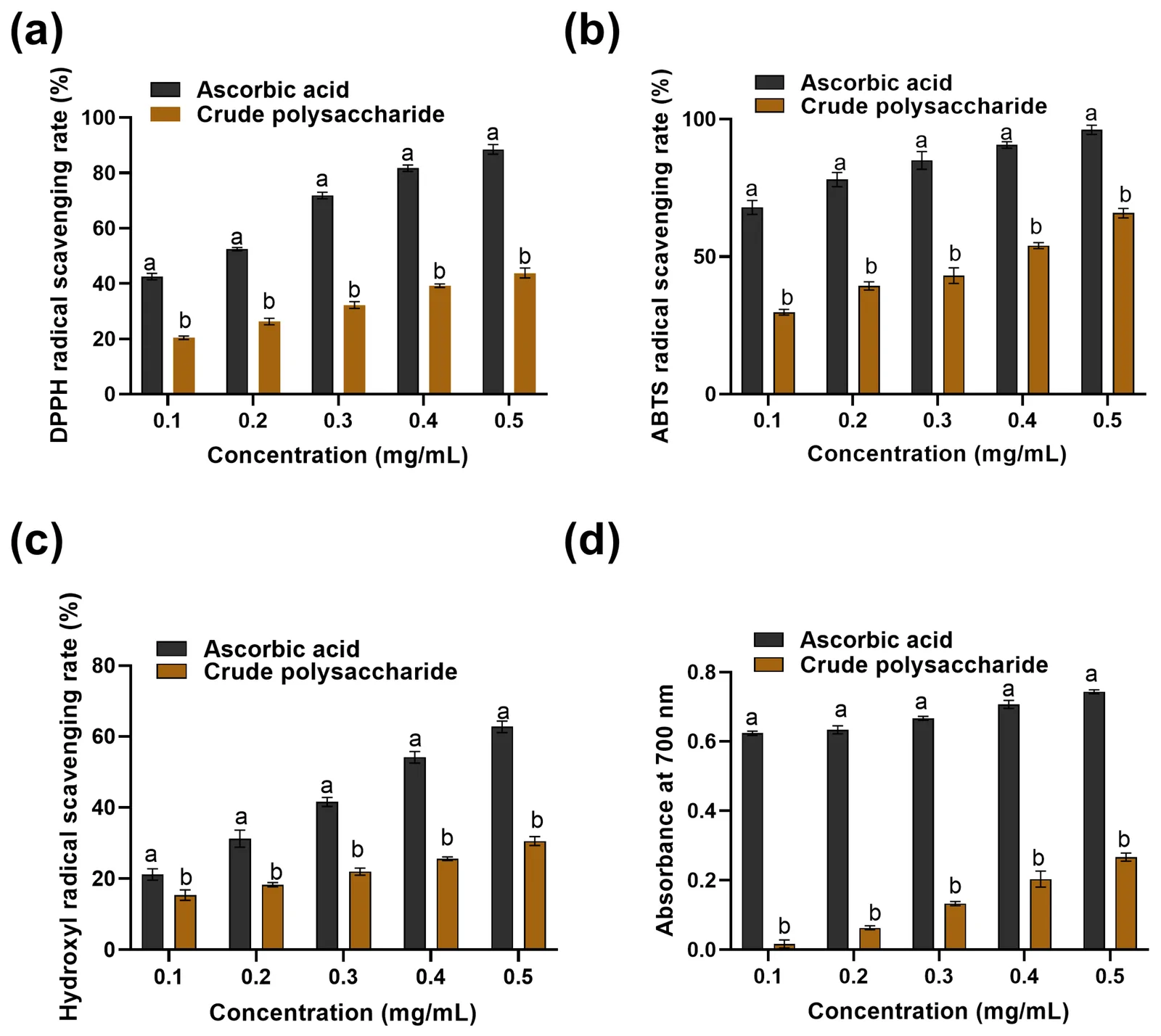
In vitro antioxidant activity evaluation of crude polysaccharides. (**a**) DPPH radical scavenging ability; (**b**) ABTS radical scavenging ability; (**c**) hydroxyl radical scavenging ability; (**d**) total reducing power (absorbance at 700 nm). All assays used ascorbic acid as the positive control, and different lowercase letters indicate significant differences between the two groups of samples at the same concentration (*p* < 0.05).

Within the tested concentration range of 0.1–0.5 mg/mL, this crude polysaccharide exhibits a significant positive concentration-dependent effect in all four classic in vitro antioxidant evaluation systems: as the sample concentration increases gradually, its free radical scavenging rate and total reducing power rise synchronously (Figure 5). This clearly confirms that the crude polysaccharide has reliable in vitro antioxidant activity, and the activity intensity enhances with the increase in sample concentration.

The DPPH free radical scavenging assay is a classic method for characterizing the quenching capacity of samples against stable nitrogen-centered free radicals. The results show that at the maximum tested concentration of 0.5 mg/mL, the DPPH free radical scavenging rate of the crude polysaccharide reaches over 40%. Although it is significantly lower than the positive control ascorbic acid, which has a scavenging rate close to 90% at the same concentration, it still reflects a moderate DPPH free radical scavenging capacity. The ABTS method can simultaneously evaluate the activity of both water-soluble and fat-soluble antioxidants, with a wider applicability. At 0.5 mg/mL, the ABTS cation radical scavenging rate of this crude polysaccharide exceeds 60%, and its activity shows a steady growth trend with increasing concentration, which verifies its effective scavenging effect on ABTS free radicals. Hydroxyl radicals are extremely oxidatively damaging reactive oxygen species in organisms, and are directly related to pathological processes such as cellular lipid peroxidation and DNA oxidative damage. The crude polysaccharide has a hydroxyl radical scavenging rate close to 30% at 0.5 mg/mL, indicating that it has a certain scavenging effect on highly toxic hydroxyl radicals and has the potential to alleviate oxidative stress in organisms. As for the total reducing power assay, the absorbance value at 700 nm directly reflects the electron-donating capacity of the sample to reduce Fe^3+^ to Fe^2+^, which is a core indicator for indirectly characterizing the total antioxidant potential. The absorbance of this crude polysaccharide gradually increases with the rise in concentration, reaching approximately 0.25 at 0.5 mg/mL, suggesting that its antioxidant mechanism is closely related to the pathways of providing hydrogen atoms and reducing active oxidative intermediates. Based on the existing five concentration gradients within 0.1–0.5 mg/mL, the DPPH scavenging rate of the crude polysaccharide does not reach 50% at the maximum tested concentration, and the ABTS scavenging rate only slightly exceeds 50% at the upper limit of the concentration range. It is impossible to obtain accurate half maximal inhibitory concentration (IC_50_) values with valid 95% confidence intervals via conventional nonlinear fitting under the current concentration setting.

At all tested concentration points, the antioxidant activity of the crude polysaccharide is significantly lower than that of ascorbic acid at the same concentration. Different lowercase letter labels at the same concentration in the figure represent statistically significant differences between the two groups of data (*p* < 0.05). This result fully conforms to the activity characteristics of natural plant crude polysaccharides: the crude extract has not been separated and purified, and the proportion of active polysaccharide components is limited, so the overall activity cannot be compared to the high-purity ascorbic acid standard. Subsequent studies can enrich the active polysaccharide components through separation and purification methods such as column chromatography to further improve the antioxidant performance of this polysaccharide and provide experimental support for its application in fields including functional food development and natural antioxidant excipients.

## 3. Materials and Methods

### 3.1. Materials, Reagents and Instruments

Fresh underground tubers of *Nephrolepis cordifolia* (L.) C. Presl were purchased from Minjian Kangle Baicao (Meizhou, China) (batch number: MJKLBC202602). The species identity was morphologically authenticated by Prof. Lihui Mou (Jiaying University) following the taxonomic criteria in Flora of China (Vols. 2–3). All reagents were of analytical grade. Rutin standard was from Shanghai Yuanye Bio-Technology Co., Ltd. (Shanghai, China); absolute ethanol and sodium hydroxide from Guangzhou Jinhua Da Chemical Reagent Co., Ltd. (Guangzhou, China); sulfuric acid and phenol from Xilong Scientific Co., Ltd. (Shantou, China); D-(+)-glucose standard, ferrous sulfate, trichloroacetic acid, ferric chloride, ammonium sulfate, potassium ferricyanide, sodium nitrite, salicylic acid, aluminum nitrate nonahydrate, ascorbic acid, ABTS from Shanghai Macklin Biochemical Co., Ltd. (Shanghai, China); DPPH from Beijing Solarbio Science & Technology Co., Ltd. (Beijing, China); potassium persulfate from Guangdong Guanghua Sci-Tech Co., Ltd. (Shantou, China). The main experimental instruments and equipment used in this study are listed as follows: 800C High-Speed Grinder (Yongkang Hongtaiyang Electromechanical Co., Ltd., Jinhua, China), BP211D Electronic Analytical Balance (Sartorius Scientific Instruments Co., Ltd., Beijing, China), INFINITE 200 PRO Full-Wavelength Microplate Reader (Tecan Trading AG, Männedorf, Switzerland), KQ3200DV Digital Ultrasonic Cleaner (Kunshan Ultrasonic Instruments Co., Ltd., Kunshan, China), and Eppendorf Centrifuge 5804R (Eppendorf AG, Hamburg, Germany).

### 3.2. Experimental Methods

#### 3.2.1. Sample Pretreatment

Fresh whole tubers of *Nephrolepis cordifolia* (L.) C. Presl were thoroughly rinsed and sliced after arrival. A two-stage drying protocol was applied: 48 h of air-drying at 25 ± 2 °C followed by 48 h of oven-drying at 60 °C to constant weight. The dried material was ground and sieved to 60–80 mesh, then stored in airtight containers at 4 °C and used within 4 months. All subsequent calculations (extraction yield, antioxidant activity) were performed on a dry weight basis.

#### 3.2.2. Extraction and Determination of Total Flavonoids

An ethanol–ammonium sulfate ATPS was pre-prepared according to the predetermined solid-to-liquid ratio. Exactly 1.0 g of *Nephrolepis cordifolia* powder was accurately weighed, added to the pre-configured ATPS, and fully homogenized. Ultrasound-assisted extraction was performed using a KQ3200DV digital ultrasonic cleaner with fixed operational parameters: 40 kHz frequency, 300 W effective output power, and a constant bath temperature of 40 ± 2 °C controlled by the integrated digital thermostat. The sample vessel was positioned at the geometric center of the ultrasonic bath to ensure homogeneous acoustic field distribution across the extraction system. The cavitation phenomenon induced by 40 kHz ultrasound generates localized micro-jets and shear forces, which rapidly disrupt the rigid cell walls of *Nephrolepis cordifolia* powder and create extensive micro-fractures on tissue surfaces, greatly enhancing the mass transfer efficiency of intracellularly bound flavonoids into the aqueous two-phase system. The mild isothermal condition avoids excessive thermal stress that would trigger covalent bond cleavage and structural transformation of thermolabile flavonoid molecules, thus maintaining their native molecular integrity, inherent antioxidant activity and characteristic physiological functions throughout the whole extraction procedure. The ultrasonic irradiation time was strictly set according to the factors defined in the experimental design. Upon completion of extraction, the mixture was allowed to stand for 30 min at ambient temperature to reach full phase equilibrium, forming a three-phase system consisting of a top ethanol-rich phase, a middle insoluble solid residue phase, and a bottom ammonium sulfate-rich aqueous phase. The upper ethanol phase enriched with target flavonoids was carefully aspirated using a pipette for subsequent processing. The collected upper ethanol phase was transferred into a pre-chilled 50 mL centrifuge tube, then centrifuged at 4000 rpm (corresponding to 2800× *g*) and 4 °C for 15 min in an Eppendorf 5804R refrigerated centrifuge to completely remove residual fine plant particulates. The exact volume of the transparent clarified supernatant was accurately recorded with a graduated pipette for subsequent extraction yield calculation, and the obtained solution was directly used as the test solution for follow-up analysis. For conventional ATPS extraction, the system adopted the identical phase composition (ammonium sulfate, ethanol, and deionized water) as the UATPE without ultrasonic assistance. The mixture was transferred to a THZ-D constant-temperature shaking incubator and agitated at 150 rpm. The extraction was performed at the same temperature (40 ± 2 °C) and for the exact same duration as the optimized UATPE process, ensuring a strictly fair performance comparison. Subsequent procedures including phase separation, target phase collection, centrifugation (4000 rpm, 15 min, 4 °C), and final volume measurement were kept completely consistent with those described for UATPE. For conventional ethanol extraction, the dried plant powder was mixed with a predefined volume of aqueous ethanol (at the concentration specified in the experimental design) following the optimized solid-to-liquid ratio. The extraction was carried out in the same constant-temperature shaking incubator under fully matched conditions: 40 ± 2 °C, 150 rpm, and the same extraction duration as the other two methods. After extraction, the mixture was centrifuged at 4000 rpm for 15 min at 4 °C, and the resulting supernatant was directly collected as the test solution for subsequent analysis. TFC was quantified via the NaNO_2_-Al(NO_3_)_3_-NaOH colorimetric assay at 510 nm, with rutin serving as the reference standard [23]. The calibration curve was linear over the range of 0–0.18 mg/mL (Y = 10.377X + 0.0064, R^2^ = 0.9991). Results are expressed as milligrams of rutin equivalents per gram of dry weight (mg RE/g DW).

#### 3.2.3. Extraction and Quantification of Crude Polysaccharides

Exactly 0.5 g of *Nephrolepis cordifolia* powder was accurately weighed into a 50 mL centrifuge tube, and an appropriate volume of distilled water was added. Ultrasonic extraction was performed using an ultrasonic cleaner. The ultrasonic extraction and separation conditions were kept identical to those used for total flavonoid extraction. After each extraction cycle, the mixture was centrifuged to collect the supernatant; this procedure was repeated for the specified number of cycles. All supernatants were pooled, and four volumes of anhydrous ethanol were added. The mixture was incubated overnight at 4 °C to precipitate polysaccharides. The precipitate was recovered by centrifugation, lyophilized, and stored as crude polysaccharide extract. Polysaccharide concentration was determined using the phenol–sulfuric acid method at 490 nm [24], with D-glucose as the calibration standard (standard curve: Y = 0.006213X + 0.002348, *R*^2^ = 0.9992; linear range: 2–200 μg/mL). The mass fraction of crude polysaccharide in the sample is calculated by the following formula: w=(C×V)∕(m×1000)×100%. Here, *w* is the mass fraction of the crude polysaccharide in the sample, %; *C* is the mass concentration of the crude polysaccharide calculated from the standard curve, μg/mL; *V* is the constant volume of the sample extract, mL; and *m* is the dry mass of the tested sample, mg.

#### 3.2.4. Construction of Ethanol–Ammonium Sulfate Phase Diagram

A 40% (*w*/*v*) aqueous ammonium sulfate solution was prepared in advance. Anhydrous ethanol was added dropwise under visual monitoring until the onset of phase separation, enabling precise determination of the critical ethanol volume required for phase transition. The phase equilibrium behavior of the ethanol–ammonium sulfate two-phase system was systematically characterized through multiple replicate experiments to construct a comprehensive phase diagram. This diagram clearly delineates the boundaries between the biphasic and monophasic regions, thereby providing a rational foundation for optimizing subsequent extraction conditions. All subsequent experimental parameters were strictly confined to the biphasic region.

#### 3.2.5. Single-Factor Experiments

For flavonoid extraction, the individual effects of four key variables on TFC were systematically investigated: extraction time (20, 30, 40, 50, and 60 min), solid-to-liquid ratio (1:20, 1:25, 1:30, 1:35, and 1:40 g/mL), ammonium sulfate mass fraction (20%, 22%, 24%, 26%, and 28%), and ethanol concentration (25%, 30%, 35%, 40%, and 45%, *v*/*v*). For polysaccharide extraction, the individual effects of three variables on the crude polysaccharide yield were systematically investigated: extraction time (10, 15, 20, 25, and 30 min), number of extraction cycles (1, 2, 3, 4, and 5), and solid-to-liquid ratio (1:10, 1:15, 1:20, 1:25, and 1:30 g/mL). All single-factor experiments were conducted in triplicate. In each trial, only one factor was varied while all other parameters were held constant. The results are presented in Figure 1 (flavonoids: a combined plot illustrating the four factors) and Figure 3 (polysaccharides: a combined plot illustrating the three factors).

#### 3.2.6. Optimization Experimental Design

For flavonoid optimization, three critical variables, namely extraction time (A), solid-to-liquid ratio (B) and ammonium sulfate mass fraction (C), were screened out as key influencing factors for flavonoid yield according to the single-factor experimental results. A three-level BBD was implemented, comprising 17 experimental runs (12 factorial points and 5 replicates at the central point). TFC served as the response variable, and a quadratic polynomial regression model was fitted using Design-Expert 13 software.

For polysaccharide optimization, three critical operational parameters were screened out from preceding single-factor experiments: extraction time (A), number of extraction cycles (B), and solid-to-liquid ratio (C), each configured at three gradient levels for the subsequent response surface design. An L_9_ (3^3^) orthogonal array was employed to systematically evaluate their effects on crude polysaccharide yield. The average yield across replicate trials was adopted as the primary response metric. Range analysis and ANOVA were conducted using IBM SPSS Statistics 27.0.0.

#### 3.2.7. Determination of Antioxidant Activity

The DPPH radical scavenging activity assay was performed following the previously reported method [25]: the optimal extracts obtained from each extraction method were prepared into a series of concentration solutions (flavonoids: 0.02–0.18 mg/mL; polysaccharides: 0.1–0.5 mg/mL). These were mixed with an equal volume of DPPH working solution (0.1–0.2 mmol/L) and incubated at 25 °C in the dark for 30 min, and the absorbance was measured at 517 nm. Ascorbic acid was used as the positive control. The ABTS radical scavenging activity assay was performed following the previously reported method [26]: ABTS stock solution was mixed with an equal volume of potassium persulfate solution and incubated at room temperature in the dark for 16 h to generate ABTS•^+^ cation radicals. The working solution was diluted with distilled water to an absorbance of approximately 0.75 at 734 nm. The sample solution was added and incubated in the dark for 6 min, and the absorbance was recorded at 734 nm. Ascorbic acid was used as the positive control. The hydroxyl radical scavenging assay was performed on the crude polysaccharides only, following the previously reported method [27]: The Fe^2+^/H_2_O_2_/salicylic acid system was used. After incubation at 37 °C in the dark for 30 min, the absorbance was measured at 510 nm. The total reducing power assay was performed on the crude polysaccharides only, following the previously reported method [28]: The potassium ferricyanide–trichloroacetic acid–ferric chloride system was used. After reaction in a 50 °C water bath, the absorbance was measured at 700 nm. This targeted approach, where the hydroxyl radical scavenging and total reducing power assays were applied exclusively to polysaccharide samples, is based on their distinct antioxidant mechanisms compared to flavonoids [20]. Polysaccharides primarily act via metal ion chelation and electron donation, which these two assays specifically evaluate, while flavonoids are more comprehensively characterized by DPPH and ABTS assays [29,30]. This design avoids redundancy and aligns with established protocols for assessing different bioactive fractions.

#### 3.2.8. Data Analysis and Statistics

RSM for flavonoid extraction optimization was performed using Design-Expert 13, with corresponding contour plots and three-dimensional response surface graphs generated via GraphPad Prism 9.5.1. For the polysaccharide orthogonal experiment, range analysis and ANOVA were conducted in IBM SPSS Statistics 27.0.0. Standard calibration curves and antioxidant activity dose–response plots were constructed using GraphPad Prism 9.5.1. All basic calculations, descriptive statistics and data summarization were completed in Microsoft Excel. Antioxidant capacity was quantified as IC_50_, derived from the nonlinear regression analysis of concentration-response curves. To enhance the statistical rigor of the dataset, 95% confidence intervals for all IC_50_ values were calculated using a 4-parameter variable-slope nonlinear regression model fitted to raw dose–response data obtained from 3 independent extraction replicates. Unless otherwise specified, all quantitative data are presented as mean ± standard deviation (SD).

Technical replicates are defined as repeated measurements of the same sample extract, including 3 parallel spectrophotometric readings for each calibration standard solution, test sample solution and blank control during the colorimetric quantification of total flavonoids and crude polysaccharides, as well as 3 parallel absorbance measurements for each concentration gradient in all antioxidant activity assays. These repeated measurements are adopted to eliminate errors introduced by instrument operation and detection procedures.

Independent extraction replicates (biological replicates) are defined as three fully independent complete extraction cycles carried out under identical experimental conditions, starting from the separate weighing of dried *Nephrolepis cordifolia* powder, through to the entire extraction, separation and purification workflow, and finally obtaining three independent batches of test samples. The single-factor experiments, response surface validation tests, orthogonal optimization verification tests, and antioxidant activity sample preparation were performed with 3 independent extraction replicates.

## 4. Conclusions

This study used the tubers of *Nephrolepis cordifolia* as the raw material and systematically optimized the extraction processes for total flavonoids and crude polysaccharides by employing UATPE combined with Box–Behnken response surface methodology and ultrasonic-assisted water extraction and alcohol precipitation combined with an L_9_ (3^3^) orthogonal design, respectively. The optimal conditions for total flavonoid extraction were an extraction time of 40.67 min, a solid-to-liquid ratio of 1:30.71 (g/mL), an ammonium sulfate mass fraction of 24.03%, and an ethanol concentration of 35%. Under these conditions, the TFC reached (54.83 ± 1.58) (mg RE/g DW), which was significantly superior to that obtained by the conventional ATPS method [(49.05 ± 0.41) (mg RE/g DW)] and the ethanol extraction method [(28.68 ± 1.54) (mg RE/g DW)]. This demonstrates that the combination of ultrasound and ATPS can substantially improve extraction efficiency. The optimal conditions for crude polysaccharide extraction were an extraction time of 10 min, three extraction cycles, and a solid-to-liquid ratio of 1:15 (g/mL). The crude polysaccharide yield was (14.89 ± 0.18) % (*RSD* = 1.21%, *n* = 3), indicating a stable and simple-to-operate process. Evaluation of antioxidant activity showed that the total flavonoids from *Nephrolepis cordifolia* exhibited strong concentration-dependent scavenging activity against both DPPH and ABTS free radicals. The flavonoids obtained by the UATPE method showed the best activity, which supports the conclusions drawn from the extraction process optimization. Within the tested concentration range of 0.1–0.5 mg/mL, this crude polysaccharide from *Nephrolepis cordifolia* exhibits a significant positive concentration-dependent activity enhancement in four classic in vitro antioxidant evaluation systems, including DPPH radical scavenging, ABTS cation radical scavenging, hydroxyl radical scavenging, and total reducing power. This result confirms its definite in vitro antioxidant capacity, and the underlying mechanism is associated with hydrogen-donating effects and the reduction of oxidative active intermediates. At the concentration of 0.5 mg/mL, the ABTS radical scavenging rate of this crude polysaccharide exceeds 60%, the DPPH radical scavenging rate reaches over 40%, the hydroxyl radical scavenging rate is close to 30%, and the absorbance value of total reducing power at 700 nm is approximately 0.25. Its overall activity is significantly lower than that of high-purity ascorbic acid at the same concentration, which is consistent with the activity characteristics of unpurified natural plant crude polysaccharides. It shows the potential to alleviate oxidative stress in organisms. Limited by the current concentration gradient setting, the existing dataset cannot support the fitting of accurate IC_50_ values that fully cover the 50% radical scavenging interval. In subsequent studies, we will supplement a series of higher concentration gradients to complete standardized IC_50_ calculation via the four-parameter Logistic model. Furthermore, we will enrich the active components through separation and purification methods such as column chromatography to improve the antioxidant performance of this polysaccharide and provide complete experimental support for its application in the development of functional foods and natural antioxidant excipients. The total reducing power assay showed a good dose-dependent relationship, confirming its in vitro antioxidant capacity in chemical-based tests. This study provides preliminary data supporting the potential value of *Nephrolepis cordifolia* as a source of bioactive compounds. The findings offer a methodological reference for the extraction of its antioxidant components. Future research should focus on the structural identification of the specific flavonoids and polysaccharides, alongside the validation of their bioactivity through cellular and in vivo models, as well as assessments of safety, bioavailability, and stability, which are essential steps prior to any consideration for functional food development.

## Figures and Tables

**Table 1:** Box–Behnken Response Surface Design Matrix.

Run	Factor A: Extraction Time (Min)		Factor B: Solid-to-Liquid Ratio (g/mL)		Factor C: Ammonium Sulfate Mass Fraction (%)		Response (Total Flavonoid Content, (mg RE/g DW))		
	Actual Value	Coded Value	Actual Value	Coded Value	Actual Value	Coded Value	Measured Value	Predicted Value	Residual
1	40	0	1:30	0	24	0	56.03	56.45	−0.4160
2	40	0	1:30	0	24	0	57.64	56.45	1.19
3	40	0	1:30	0	24	0	55.98	56.45	−0.4660
4	40	0	1:35	1	26	1	46.27	46.19	0.0775
5	50	1	1:30	0	22	−1	41.93	42.01	−0.0787
6	30	−1	1:35	1	24	0	41.31	41.47	−0.1562
7	40	0	1:25	−1	26	1	42.61	42.90	−0.2900
8	30	−1	1:30	0	26	1	40.16	40.08	0.0788
9	30	−1	1:25	−1	24	0	39.13	38.92	0.2113
10	50	1	1:30	0	26	1	42.72	42.59	0.1338
11	40	0	1:30	0	24	0	56.29	56.45	−0.1560
12	50	1	1:35	1	24	0	44.33	44.54	−0.2112
13	50	1	1:25	−1	24	0	40.65	40.49	0.1563
14	30	−1	30	0	22	−1	39.73	39.86	−0.1337
15	40	0	25	−1	22	−1	42.42	42.50	−0.0775
16	40	0	35	1	22	−1	46.09	45.80	0.2900
17	40	0	30	0	24	0	56.29	56.45	−0.1560

**Table 2:** ANOVA for the Response Surface Regression Model of Total Flavonoid Extraction from *Nephrolepis cordifolia*.

Source	Sum of Squares	df	Mean Square	*F*-Value	*p*-Value
Model	767.58	9	88.29	267.60	<0.0001 **
A-Extraction time	10.81	1	10.81	33.92	0.0006 **
B-Solid-to-liquid ratio	21.75	1	21.75	68.23	<0.0001 **
C-Ammonium sulfate mass fraction	0.3160	1	0.3160	0.9915	0.3525
AB	0.5625	1	0.5625	1.76	0.2257
AC	0.0324	1	0.0324	0.1017	0.7592
BC	0.0000	1	0.0000	0.0001	0.9932
A^2^	352.65	1	352.65	1106.48	<0.0001 **
B^2^	148.53	1	148.53	466.01	<0.0001 **
C^2^	159.73	1	159.73	501.18	<0.0001 **
Residual	2.23	7	0.3187	-	-
Lack of Fit	0.3665	3	0.1222	0.2621	0.8500
Pure Error	1.86	4	0.4661		
Cor Total	769.82	16			

Note: *R*^2^ = 0.9971, adjusted *R*^2^ = 0.9934, *CV* = 1.22%; ** denotes highly significant difference at *p* < 0.01.

## Data Availability

The original contributions presented in this study are included in the article. Further inquiries can be directed to the corresponding authors.
